# A Real-Time Collision Avoidance Framework of MASS Based on B-Spline and Optimal Decoupling Control

**DOI:** 10.3390/s21144911

**Published:** 2021-07-19

**Authors:** Xinyu Zhang, Chengbo Wang, Kwok Tai Chui, Ryan Wen Liu

**Affiliations:** 1 Navigation College, Dalian Maritime University, Dalian 116026, China; wangcb@dlmu.edu.cn; 2 School of Science and Technology, The Open University of Hong Kong, Hong Kong; jktchui@ouhk.edu.hk; 3 Hubei Key Laboratory of Inland Shipping Technology, School of Navigation, Wuhan University of Technology, Wuhan 430063, China; wenliu@whut.edu.cn

**Keywords:** collision avoidance, maritime autonomous surface ships, B-spline, optimal decoupling control, real-time

## Abstract

Real-time collision-avoidance navigation of autonomous ships is required by many application scenarios, such as carriage of goods by sea, search, and rescue. The collision avoidance algorithm is the core of autonomous navigation for Maritime autonomous surface ships (MASS). In order to realize real-time and free-collision under the condition of multi-ship encounter in an uncertain environment, a real-time collision avoidance framework is proposed using B-spline and optimal decoupling control. This framework takes advantage to handle the uncertain environment with limited sensing MASS which plans dynamically feasible, highly reliable, and safe feasible collision avoidance. First, owing to the collision risk assessment, a B-spline-based collision avoidance trajectory search (BCATS) algorithm is proposed to generate free-collision trajectories effectively. Second, a waypoint-based collision avoidance trajectory optimization is proposed with the path-speed decoupling control. Two benefits, a reduction of control cost and an improvement in the smoothness of the collision avoidance trajectory, are delivered. Finally, we conducted an experiment using the Electronic Chart System (ECS). The results reveal the robustness and real-time collision avoidance trajectory planned by the proposed collision avoidance system.

## 1. Introduction

As for maritime autonomous surface ships (MASS), the sea is vast and infinite, and the navigation situation information perception is very limited. Meanwhile, there is uncertainty in the accuracy of the prediction of obstacle motion and collision trajectory [1]. The ability to autonomously navigate in this uncertain environment can undoubtedly greatly improve the properties of safety and effectiveness for MASS. The collision avoidance algorithm is a crucial element of autonomous navigation, making strategies to avoid all obstacles and achieve collision-free. To fulfill the need of practice, collision avoidance must be real-time, optimal in duration and control cost, dynamically feasible, and path smooth, to guarantee safe and effective autonomous navigation.

At present, most of the theories of collision-avoidance navigation are however focused on collision avoidance decision-making or planning. For collision avoidance decision-making or planning theories, there is mainly a divide into four kinds of methods, such as obstacle avoidance path planning based on an intelligent search algorithm [2,3], local path planning based on optimization theory [4], collision avoidance decision-making based on adaptive algorithm [5], and obstacle avoidance control method [6]. However, these methods only study the problem of collision avoidance decision-making or obstacle avoidance control individually, without fully considering the coupling of decision and control. With the further research of automatic navigating, unmanned surface vehicles (USV) technology has become a research hotspot, and has been applied in various practical fields, such as environmental monitoring, sampling, and coastal surveying, and mapping [7].

Due to the large tonnage, slow speed, weak maneuverability, large inertia and other typical underactuated characteristics of autonomous cargo ships, the technology of quick control and dexterous speed change of unmanned surface vehicles cannot be applied to MASS [8]. Therefore, for MASSs, it is necessary to decouple navigation from collision avoidance mission planning, decision-making, and optimal decoupling control. However, the coupling of collision avoidance and optimal control has rarely been studied directly in the field of autonomous ships.

To fill the above-mentioned gap, this study proposes a real-time collision avoidance framework based on optimal decoupling control and B-spline. Through the verification in several scenes, the proposed algorithm yields superior performance in real-time collision avoidance. In addition, our framework has also been used in the Information Display System of Collision Avoidance Support Decision, and Intelligent Collision Avoidance System. The research contributions of this article are summarized as below.

- A BCATS algorithm is proposed to decide a safe dynamically feasible, and reasonable collision avoidance strategy. The performance evaluation of the proposed framework is confirmed by several simulation experiments.
- A velocity-path decoupling-based control waypoints optimization is proposed to reduce the control cost, and improve the smoothness of the real-time collision avoidance framework.
- The algorithm and framework proposed in this article are integrated into the Information Display System of Collision Avoidance Support Decision, which is the key part of the Integrated bridge system (IBS).

The remainder of the paper is organized as follows. Section 2 presents related works. The overall structure of the real-time collision avoidance framework is introduced in Section 3. Section 4 presents the methodology of the B-spline and optimal decoupling control. The verification and case study are carried out in Section 5. Finally, a conclusion is drawn.

## 2. Related Works

In this section, a literature review about collision avoidance for autonomous ships was conducted, for foreign and domestic routes. The qualitative research on collision avoidance of ships at sea began in the decade after World War II [9]. The research on ship collision avoidance mainly benefits from the progress of industrial radar and the increase of shipping trade. Until the 1990s, many scholars and experts began to consider and use computer means, soft computing, and other technologies to study collision avoidance algorithms to address the issue of multi-ship collision [10]. The collision avoidance methods include velocity obstacle method (VO) [11], artificial potential field (APF) [12], A-Star [13], rapidly exploring random tree (RRT) [14,15,16], genetic algorithm [17], fuzzy theory [18], deep reinforcement learning (DRL) [19], and spline curves [20]. But overall, it can be classified into four categories, such as traditional algorithms, soft computing algorithms, intelligent learning algorithms, and spline curves.

In terms of traditional algorithms, Wang et al. [21] presented a design for MASS using an innovative collision avoidance decision-making method, which consisted of the front end (presentation layer) and back end (data access layer). The former detects collision risk and juggles the encounter situation whereas the latter generates a collision avoidance strategy. It is worth mentioning that the collision avoidance method of this reference introduced various constraints, such as ship maneuverability, Convention on the International Regulations for Preventing Collisions at Sea 1972 (COLREGs), and seamanship, using a modified VO algorithm. Lyu et al. [22] proposed a real-time modified APF-based obstacle avoidance path planning approach for USV in complex and dynamic navigation environments. It was embedded in the ship navigation simulator to verify the function of planning and obstacle avoidance. Chen et al. [23] proposed an improved A-Star-based meteorological route planning algorithm. It is used to optimally design the convex and concave obstacle environments to realize the shortest path. The search path nodes are reduced. For the above-mentioned collision avoidance and obstacle avoidance path planning algorithms, most of the simple “avoidance” and “shortest” principles of obstacle avoidance and navigation path planning cannot be practical.

For soft computing and intelligent computing algorithms, Lazarowska [24] utilized an ant colony algorithm to realize path planning in a dynamic environment. This method can be used in ship obstacle avoidance as a decision support system and USV navigation system. A hybrid genetic algorithm was introduced to enhance the theoretical safe-critical collision avoidance trajectory planning algorithm to align with good seamanship and COLREGs [25]. For the bionic algorithm represented by the particle swarm optimization algorithm, the genetic algorithm, the ant colony algorithm, etc., most of them were adopted to search for the globally optimal path. However, these algorithms are limited to fulfill the real-time requirements. Ahn et al. [26] integrated a neural network with collision risk calculation, with a fuzzy inference system and expert system to achieve ship collision avoidance. It can be well applied in the actual navigation system of ships. However, due to the limited number of input and output values, this method loses some accuracy. On the other hand, the processing speed of continuous reasoning is slow which is a challenge to ensure the completeness of the expert system. To avoid ship collision in multi-ship encounter situations, Liu et al. [27] proposed a fuzzy-neural network algorithm. Liu et al. [3] proposed a hybrid fast marching method (FMM) and self-organizing map (SOM) algorithm for obstacle avoidance path planning, which combined mission planning and collision avoidance.

Some other methods applied a deep learning/deep reinforcement learning (DRL) or a reinforcement learning (RL) algorithm into ship collision avoidance decision-making or obstacle avoidance control. Generally, deep learning is used for target recognition or obstacle recognition in the early stage of collision avoidance [28]. A DRL-based COLREGs-compliant algorithm was proposed for multi-ship collision avoidance [29]. Sawada, et al. [30] extended the DRL for continuous action spaces using an automatic collision avoidance algorithm. Researchers redesigned the long short-term memory (LSTM) network and trained the model in continuous action spaces. Results demonstrated an improvement in safe distance compared with previous works. In [6], the issue of low efficiency in the model-free RL algorithm under an unknown environment was raised. The feed-LSTM controller and Q-learning are combined to generate supervised trajectories for the Actor-Critic Algorithm (A3C).

However, these ship collision avoidance approaches have shared common limitations. In the existing works, the front end of the system usually plans the obstacle avoidance and planning on collision avoidance by considering of collision avoidance mission, while ignoring the performance of ship dynamics. In view of the maneuverability of ships, some scholars have studied the obstacle avoidance control methods based on considering COLREGs [31,32]. In the obstacle avoidance planning and collision avoidance decision-making of the front-end, existing methods usually only consider the collision avoidance mission but ignore the performance of ship dynamics. For existing methods of ship collision avoidance control, back end obstacle avoidance trajectory optimization often takes a long iteration time.

To summarize, for a collision avoidance algorithm applied to MASS, dynamically feasible, highly reliable, and safe collision avoidance are key standards for application and evaluation. The spline curves method can achieve this function of collision avoidance for MASS. In recent years, spline curves are mainly used in robots, unmanned vehicles, and aircraft. Choi et al. [33] proposed a trajectory planning of a dual-arm robot based on a B-spline curve to realize a collision-free path. In [34], a B-spline curves approach was proposed to obtain a collision-free path.

To tackle the limitations of the existing methods, a dynamic collision avoidance algorithm based on B-spline and optimal decoupling control with real-time was proposed. Different from previous methods, in the front-end, the mathematical model of MASS’s autonomous collision avoidance decision-making with the minimum total navigation time as the objective function is constructed to solve the ship’s control waypoint and ship’s deduction trajectory. The back end uses the path-speed decoupling optimal control.

## 3. Framework Overview

The collision-avoidance navigation system, as the most direct embodiment of a ship’s intelligence, is a crucial element of the MASS. Firstly, the system obtains the preset destination coordinates and the data transmitted by the sensing system, including the ship’s position, ship speed, course, relative orientation, relative distance, and other information. Then, it models, predicts, and understands the intention of the target ship, outputs a series of global planning routes composed of control steering points, and guides the ship to the destination by controlling the steering points. If there are unknown obstacles or the original route is blocked in the process of navigation after planning, the collision-avoidance navigation system will carry out secondary planning and re-plan a reasonable and optimal derived route.

**Figure 1:**
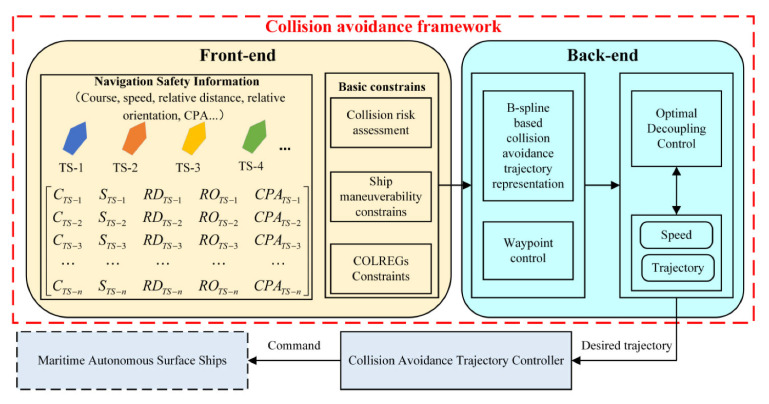
The framework of autonomous navigation system for MASS, collision avoidance included. In Figure 1, C is the course, S is the speed, RD is the relative distance from the own ship to the target ship, RO is the relative orientation between the own ship and the target ship, CPA is the closest point of approach between the own ship and the target ship, TS−n is the nth target ship, and n is a positive integer, and 0.

Here we show the autonomous navigation system framework for MASS in Figure 1. The proposed collision avoidance framework, indicated in the red dotted box, can serve as a planning module. The framework is divided into two parts, the front end and the back end. The front end is mainly responsible for the perception of navigation safety information of all encounter situations. Then, the front end needs to evaluate the risk of collision between the own ship and target ships. In the front end of the framework, the system also considers ship maneuverability constraints and COLREGs constraints before collision avoidance decision-making. The back end mainly includes B-spline based collision avoidance trajectory representation, waypoint control, and path-speed optimal decoupling control. Finally, the optimal action on collision avoidance is delivered to the ship collision avoidance trajectory controller system, to achieve the free-collision navigation of MASS. The details of the collision avoidance framework and method are referred to in Section 4.

## 4. Collision Avoidance Method

The purpose of this paper is to realize the path-speed decoupling of MASS, and to ensure that the ship meets collision risk assessment, ship maneuverability constraints, and COLREGs constraints within the planning time, which is transformed into the problem of optimal control. Finally, it realizes intelligent collision avoidance in the dynamic and static uncertain environment.

### 4.1. Basic of Collision Avoidamce

#### 4.1.1. Collision Risk Assessment

The ship collision risk index is obtained by quantifying ship collisions. This index can be used to measure the risk of ship collision. At the same time, it is also the basis and evaluation standard for making collision avoidance decisions. The risk of ship collision is measured by the time to the closest point of approach (TCPA) and the distance to the closest point of approach (DCPA). The main symbols about collision risk assessment are shown in Table 1:

DCPA and TCPA are as follows.

(1) $$DCPA=RD*\sin(Q_{r})$$

(2) $$TCPA=\frac{RD*\cos(Q_{r})}{v_{r}}$$

Therefore, the collision risk of ships is as follow [35].

(3) $$R=a\;[e^{{\left(\frac{DCPA}{D_{b}}\right)}^{2}\cdot b}-0.1][\frac{T_{b}}{TCPA+c}-d]$$

where a, b, c, and *d* are the regulated parameters.

#### 4.1.2. Ship Maneuverability Constraints

Both the own ship and the target ship (TS) will be simplified as the same circle. The ship’s course and speed can be mapped to the variation of lateral velocity and longitudinal velocity. By limiting the ship’s lateral speed, longitudinal speed, and waypoints, the ship’s course, speed, and acceleration can be indirectly controlled. Finally, the ship collision avoidance control is realized considering the constraints of the ship kinematics model.

Three-degree-of-freedom ship motion is considered, i.e., surge, sway, and yaw; the ship maneuverability constrained equations can be conveniently expressed as [36]:

(4) $$M{\dot{v}}_{r}+C(v_{r})v_{r}+D(v_{r})v_{r}+g(\eta )+g_{0}=\tau +\tau_{wind}+\tau_{wave}$$

where M, $C(v_{r})$, $D(v_{r})$, g(η), $g_{0}$, τ, $\tau_{wind}$, and $\tau_{wave}$ are the system inertia matrix, the Coriolis-centripetal matrix, the damping matrix, the vector of gravitational/buoyancy moments and forces, the vector used for pre-trimming, the vector of control inputs, the vector of wind forces, and the vector of wave-inducted forces, respectively.

**Figure 2:**
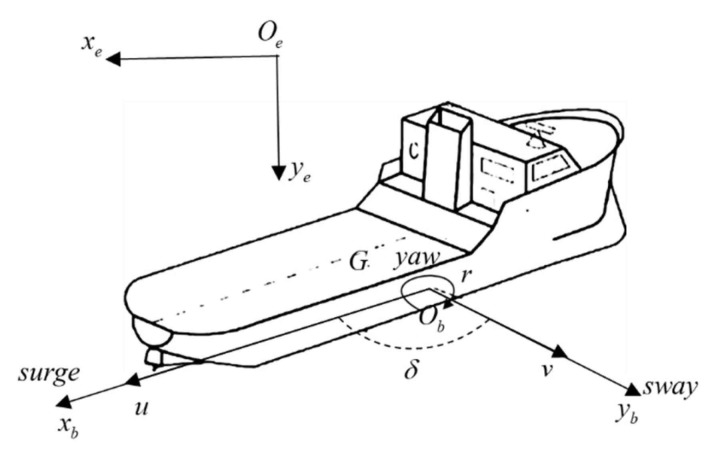
The coordinate systems of the MASS. The $X_{e}O_{e}Y_{e}$ represents the geodetic coordinate system. The $X_{b}O_{b}Y_{b}$ represents the horizontal ship body-fixed coordinate system. G is the center of ship gravity, u is longitudinal velocity, v is lateral velocity, δ is yaw angle, and r is the yaw angular velocity.

Figure 2 presents the coordinate systems. It can be seen from the architecture that the ship collision avoidance problem can be transformed into *surge*, *sway*, and *yaw*, like the navigation motions.

Thus, the horizontal plane motion equations of the MASS are defined as follows.

(5) $$\left\{\begin{array}{c} \dot{u}=X/m+vr\\ \dot{v}=Y/m-ur\\ \dot{\delta }=r\\ \dot{r}=N/I_{ZZ}\\ {\dot{x}}_{OS}=u\cos\delta -v\sin\delta \\ {\dot{y}}_{OS}=v\cos\delta +u\sin\delta \end{array}\right.$$

where $x_{OS}$ is longitude of the own ship’s position and $y_{OS}$ is the latitude of the own ship position.

The expressions of physical quantities of various motion forms in Equation (5) are shown in Table 2.

#### 4.1.3. COLREGs Constraints

Before the B-spline based collision avoidance trajectory representation is calculated, the COLREGs must be considered. Although it is not known whether the existing COLREGs are applicable to MASS, IMO is also promoting the change of rules to apply to the coexistence of MASS and traditional manned ships. In this paper, we ignored the collision-avoidance action of target ships. We assume that target ships keep the direction and speed. Therefore, we mainly applied rules 13–15 of COLREGs.

**Figure 3:**
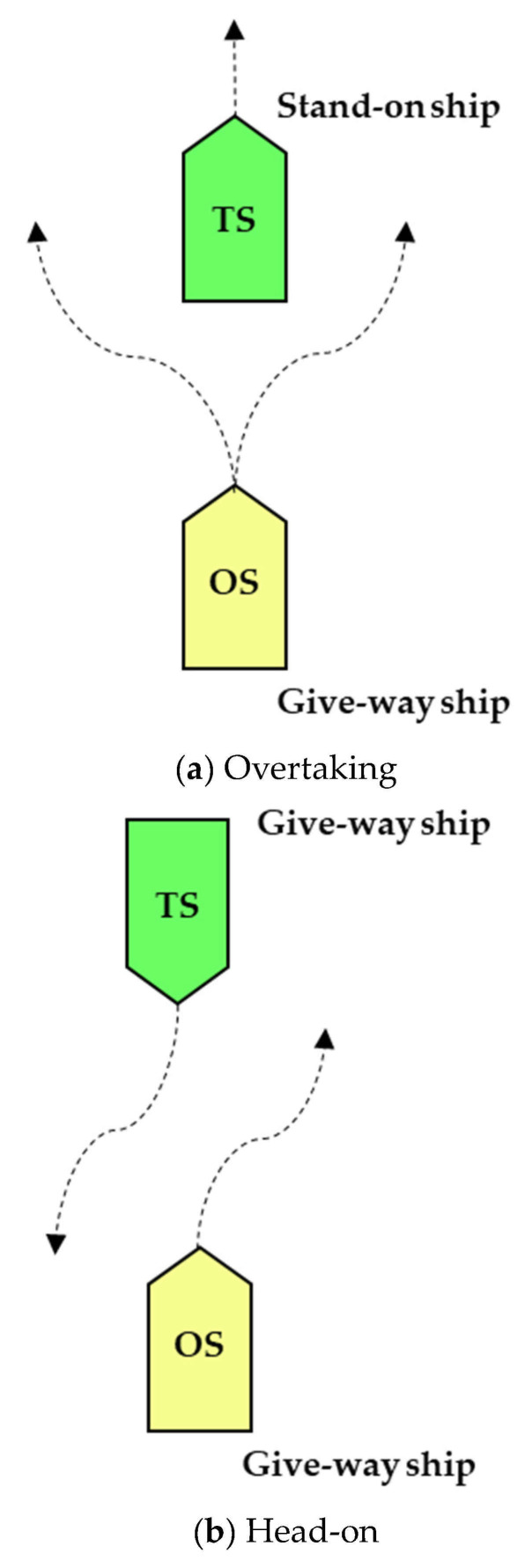

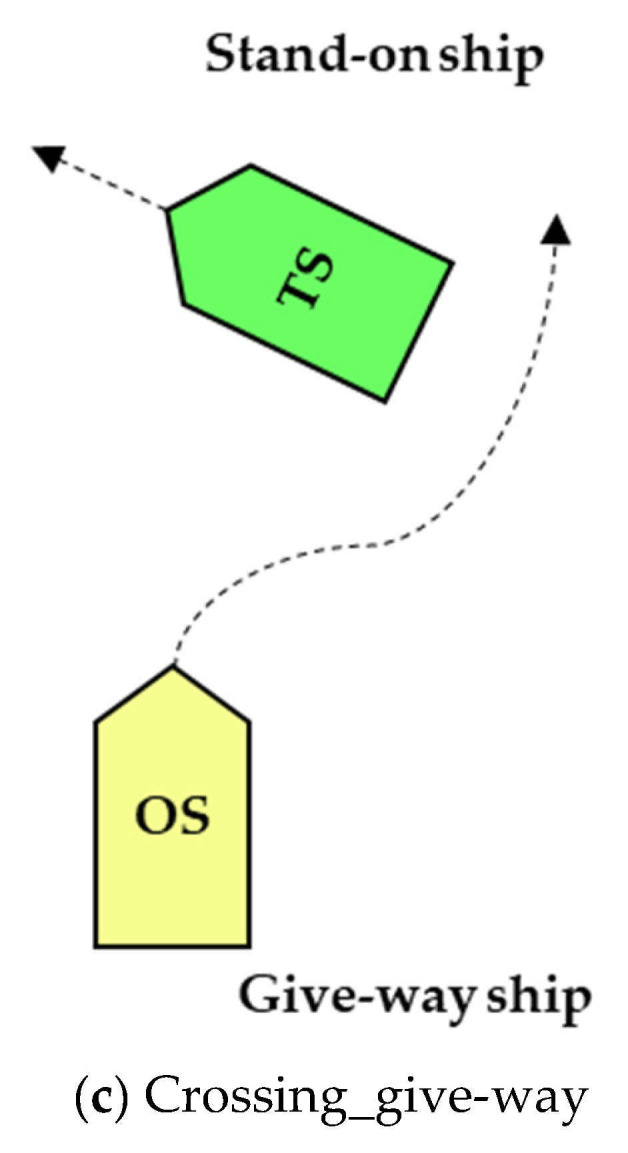
Three encounter situations divided by COLREGs: (**a**) Overtaking, (**b**) Head-on, and (**c**) Crossing_give-way.

In other words, the encounter situation between the own ship and targets ships is divided into the following three types: (a) Overtaking, (b) Head-on, and (c) Crossing_ give way, which is shown in Figure 3. In this paper, we assume that target ships are keeping their course and speed at all voyages, and we add rules to the initial planning before collision avoidance trajectory representation based on B-spline. Specifically, the behavior of avoiding obstacles based on COLREGs is mainly left turn and right turn. Finally, the collision avoidance trajectory and waypoints are calculated based on the BCATS algorithm.

### 4.2. B-Spline Based Collision Avoidance Trajectory Representation

The B-spline curve is an improved version of the Bezier curve. Through a Bezier curve, a complex shape curve can be drawn. As long as the points representing the general trend of the curve are given as control turning points, a control polygon can be drawn from these points, and then the desired curve can be drawn by approximating the polygon through the Bezier formula.

In 2D space, like collision avoidance on the maritime surface, the B-spline curve value can be defined as the following Equation (6).

(6) $$P(t)=\sum_{i=0}^{n}p_{i}B_{i,k}(t),\;t_{\min}\leq t\leq t_{\max}$$

where $p_{i}\in {\mathbb{R}}^{2},i\in \left\{0,1,2,\cdots ,m\right\}$ are the control waypoints corresponding to $t_{i}$. $B_{i,k}(t)$ are B-spline basis functions. The i−th B-spline basis function of degree p is defined, in a recursive manner, as:

(7) $$B_{i,0}(t)=\left\{\begin{array}{c} 1,\;if\;t_{i}\leq t\leq t_{i+1}\\ 0,\;\mathrm{otherwise} \end{array}\right.$$

(8) $$B_{i,p}(t)=\frac{t-t_{i}}{t_{i+p}-t_{i}}B_{i,p-1}(t)+\frac{t_{i+p+1}-t}{t_{i+p+1}-t_{i+1}}B_{i+1,p-1}(t),p>0$$

**Figure 4:**
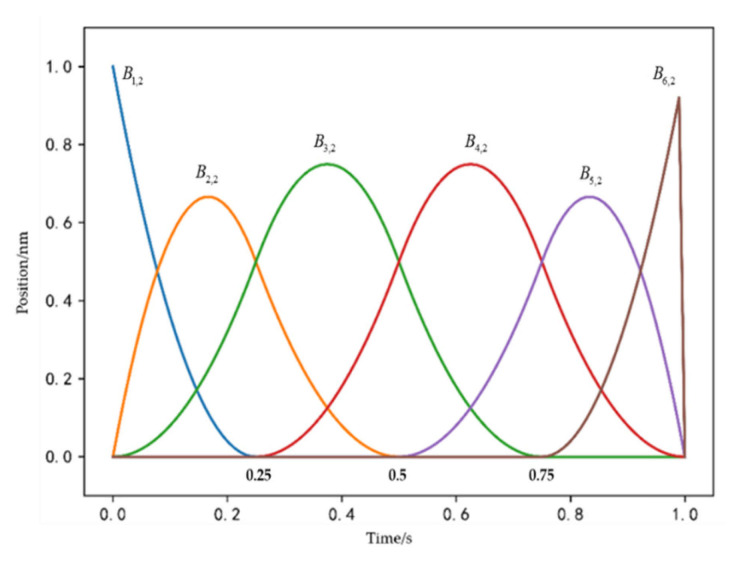
Quadratic univariate B-spline basis functions (polynomial degree *p* = 2).

Figure 4 shows quadratic univariate B-spline basis functions defined by the uniform and open knot vector. In this paper, uniform and open knot vectors are used. B-spline basis functions, as well as control waypoints, can be obtained by the standard knot refinement and degree elevation from the initial knot vector $\left\{0,0,1,1\right\}$. From Figure 4, we can see the uniform and open knot vector of quadratic univariate B-spline basis functions (*p* = 2) is $\left\{0,0,0,0.25,0.5,0.75,1,1,1\right\}$.

In order to meet the above boundary conditions of optimal control for a collision avoidance point, the B-spline curve is utilized to parameterize the position information, speed information, and heading information of the own ship (OS) and the TS. The position information is parameterized to Equation (9).

(9) $$P_{OS}(t)=\left[\begin{array}{c} x_{OS}\\ y_{OS} \end{array}\right]$$

where $x_{OS}$ and $y_{OS}$ are the longitude and latitude of the position of the OS, respectively.

(10) $$x_{OS}(t)=\sum_{i}^{n}p_{i}^{x}B_{i}(t)$$

(11) $$y_{OS}(t)=\sum_{i}^{n}p_{i}^{y}B_{i}(t)$$

Then, we can turn a<p(t)<b into $a<p_{i}<b$. As can be seen from Figure 4, removing the initial knot vector $\left\{0,0,1,1\right\}$, the maximum degree of B-spline basis function is 0.75.

(12) $$p_{i}\leq 0.75,\;\forall t\in [0,T]$$

**Figure 5:**
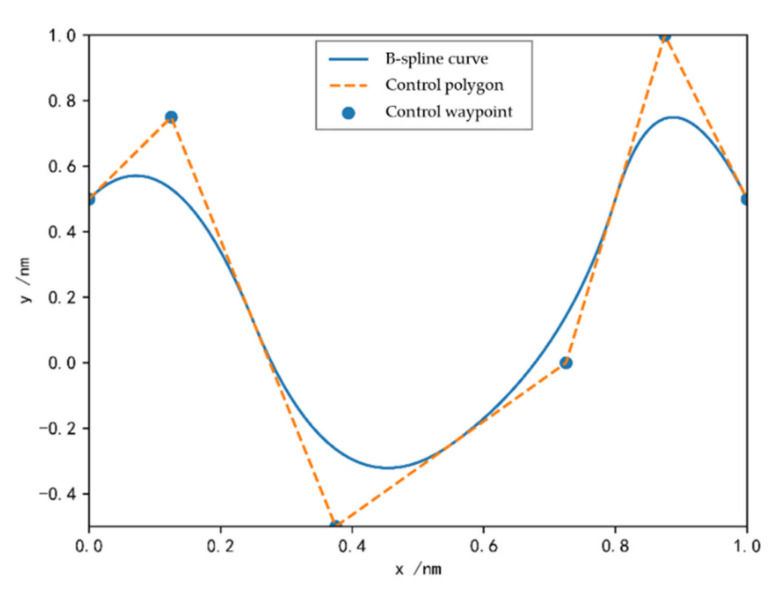
B-spline and its control polygon.

Therefore, the position information of collision avoidance trajectory can be illustrated with Figure 5.

In this paper, we set the start position and target position. Then, by limiting the control waypoints to restrict the ship collision avoidance trajectory we can control the speed, acceleration, and steering angle to achieve the represent the free-collision trajectory:

(13) $$\left\{\begin{array}{c} p(0)=p_{0}\\ p(T)=p_{goal}\\ V_{\min}\leq V(t)\leq V_{\max}\\ {\dot{V}}_{\min}\leq \dot{V}(t)\leq {\dot{V}}_{\max}\\ \gamma_{\min}\leq r(t)\leq \gamma_{\max} \end{array}\right.$$

where $p_{0}$, $p_{goal}$, $V_{\min}$, $V_{\max}$, ${\dot{V}}_{\min}$, ${\dot{V}}_{\max}$, $\gamma_{\min}$, and $\gamma_{\max}$ are the starting point, the target point, the minimum speed, the maximum speed, the minimum acceleration, the maximum acceleration, the minimum steering angle, and the maximum steering angle of the OS, respectively.

### 4.3. Path-Speed Decoupling Optimal Control

**Figure 6:**
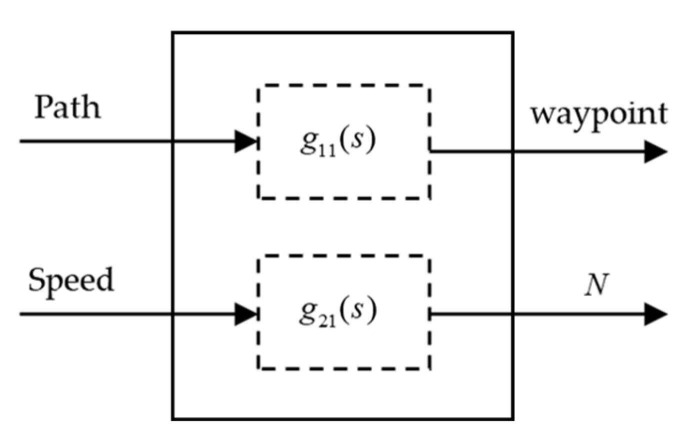
Path-speed decoupling system. *N* represents the ship thrust output; $g_{11}(s)$ and $g_{21}(s)$ represent the controller of waypoint and thrust, respectively.

For MASS, since the speed and path (heading) are independent, single input single output control can be applied to these two aspects respectively. The schematic diagram of the decoupling system is shown in Figure 6. Ship collision avoidance motion is coupled in speed and path. Each output variable of the multivariable ship obstacle avoidance motion control system, which is composed of path and speed, is completely controlled by only one input variable, and different outputs are controlled by different inputs. After decoupling, the cross-coupling between input and output variables is removed, and the waypoint and thrust are controlled autonomously, that is the control without mutual influence.

#### 4.3.1. Collision Avoidance Trajectory Planning and Waypoint Control

The collision avoidance trajectory planning sub-module can express the collision-free path of the MASS as the target ship or obstacle without covering. For this reason, this paper proposes to use the separation theory of hyperconvex sets to build the decision-making model of ship collision avoidance. It is worth noting that the concept of two disjoint convex sets can be regarded as the part where two convex sets do not intersect or overlap. Therefore, a hyperplane can be used to separate the two convex sets. After expansion, multiple convex sets can be separated. In the 2D plane, two disjoint convex sets can always be separated by a hyperplane, which transforms the ship collision avoidance problem into binary classification.

Assuming that the ship and obstacle are simplified as a circle, the collision avoidance problem is described as Equation (14).

(14) $$\mathrm{distance}(p_{OS}(t),p_{TS}(t))>2\varsigma$$

where $p_{TS}(t)$ is the position of TS at time t, $p_{OS}(t)$ is the position of OS at time t, $\mathrm{distance}(p_{OS}(t),p_{TS}(t))$ is the distance between OS and TS, and ς is the radius of the field of ship safety, respectively.

To fulfill the above conditions, the hyperplane parameters are spline treated. The equations of a hyperplane in the 2D Cartesian coordinate system are as follows.

(15) ax+by+c=0

where (a,b) is the normal vector of hyperplane and c is the offset of the hyperplane.

When it is expressed as a straight line on the two-dimensional plane, it should be ensured that the safe distance of the ship is less than the separation distance between the ship and the hyperplane. The specific treatment method is shown below.

(16) $$[a(t),b(t)]\left[\begin{array}{c} x\\ y \end{array}\right]-c(t)>d+\varsigma$$

where d is the shortest distance of safety navigation for MASS.

In order to make Equation (16) controllable, it is necessary to satisfy the constraint that the modulus of the normal vector of the hyperplane is less than 1.

(17) $$\left\{\begin{array}{c} \sqrt{a^{2}+b^{2}}<1\\ \forall t\in [0,T] \end{array}\right.$$

In the process of ship motion, the normal vector and offset of the hyperplane are parameterized by spline to obtain the hyperplane.

(18) $$\begin{array}{l} a(t)=\sum_{i}^{n}P_{i}^{a}B_{i}(t)\\ b(t)=\sum_{i}^{n}P_{i}^{b}{}_{i}B_{i}(t)\\ c(t)=\sum_{i}^{n}P_{i}^{c}B_{i}(t) \end{array}$$

**Figure 7:**
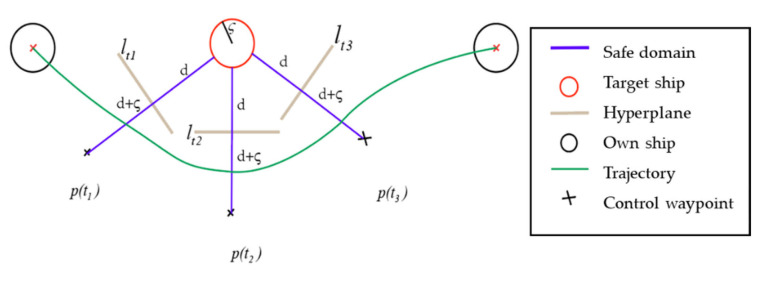
Collision avoidance trajectory planning and waypoint control.

In the dynamic environment, this paper assumes that the target ship is in the situation of keeping direction and speed. The model predicts the position of the ship and TS at each moment so that a control turning point can be determined in each moment. Refer to Figure 7, at $t_{2}$, the ship predicts that the TS hinders the ship’s progress, so the ship solves the hyperplane by optimizing the objective function in real time. In the figure, $p(t_{1})$ and $p(t_{2})$ are the turning points to avoid the target ship, and $p(t_{3})$ and $p(t_{n})$ are the turning points to advance to the target point.

#### 4.3.2. Optimal Decoupling Control Solving

The proposed path-speed decoupling algorithm utilized the optimal control algorithm to solve the model. The speed planning of MASS is transformed into path planning. Therefore, this paper only solves the collision avoidance trajectory planning of the MASS. The best navigation path with static and dynamic obstacles can be obtained for the real-time collision avoidance system, under an uncertain environment.

The 2D collision avoidance trajectory of the MASS is a B-spline curve in 2D space, which can be represented by a set of control waypoint sequences. This set of control waypoint sequences is the output of the BCATS algorithm. Therefore, the optimization variable is the sequence of control waypoints $\left\{p_{1},p_{2},\cdots ,p_{n}\right\}$. The purpose of collision avoidance trajectory optimization is further to reduce the control cost and take the shortest time to avoid target ships. We turn the total control cost of a set of control waypoint sequences into the total time-consuming problem. So, the optimization goal is defined as follows:

(19) $$\min T^{c}=\sum_{i=0}^{n}\frac{{\left\|dP(t)\right\|}^{2}}{\int_{t_{i}}^{t_{i+1}}\omega {\left(\frac{dP(\dot{V}(t)-i)}{d^{\prime }t}\right)}^{2}dt}$$

where $T^{c}$ is the total time-consuming, ω is the weight, P(t) is the trajectory value of B-spline curve at time t, and $\dot{V}(t)$ is the acceleration at time t.

The collision avoidance trajectory optimization method is described in this section. We consider a variety of constraints. Therefore, free-collision requirements and dynamic constraints need to be added to optimization variables. In particular, the following linear inequality constraints are obtained:

(20) $$\begin{array}{l} \left\{\begin{array}{l} p(0)=p_{0}\\ p(T)=p_{T}\\ V_{\min}\leq V(t)\leq V_{\max}\\ {\dot{V}}_{\min}\leq \dot{V}(t)\leq {\dot{V}}_{\max}\\ \gamma_{\min}\leq \gamma (t)\leq \gamma_{\max}\\ {}[a(t),b(t)]\left[\begin{array}{c} x\\ y \end{array}\right]-c(t)>d+\varsigma \\ \sqrt{a^{2}+b^{2}}<1\\ \forall t\in [0,T] \end{array}\right. \end{array}$$

In this paper, the minimum navigation time T is taken as the goal, and the starting point, target point, speed, acceleration, steering rate, and the safe distance between the TS and the ship are limited. The autonomous collision avoidance model of MASS is constructed. By parameterizing the time t, all constraints can be applied to the ship kinematics model. The B-spline curve can parameterize the ship motion model, which can further be applied to the ship autonomous collision avoidance problem.

## 5. Case Study and Discussion

The proposed collision avoidance algorithm and framework have been successfully applied to the Information Display System of Collision Avoidance Support Decision based on the Electronic Chart System (ECS). There are two main parts to the experimental verification platform:

1. Ship autonomous collision avoidance decision-making platform: based on the Ubuntu system, using the CasADi module of Python modeling;
2. Collision avoidance display platform based on the electronic chart: Windows system, based on the Tianjin Port Electronic Chart platform.

In this section, we set up 5 different scenarios to test and verify the algorithm and method. According to the actual situation of ship navigation, the main verification scenarios we designed are overtaking, head-on, crossing, multi-ship encounter into open water, and multi-ship encounter in a busy sea area.

### 5.1. Scenario 1: Overtaking

In this simulation experiment, there is a target ship keeping direction and speed, and the ship needs to cross the dangerous sea area to reach the target point. Table 3 shows the initial information (including position, course, speed, RD, and bearing) of the OS and TS.

**Figure 8:**
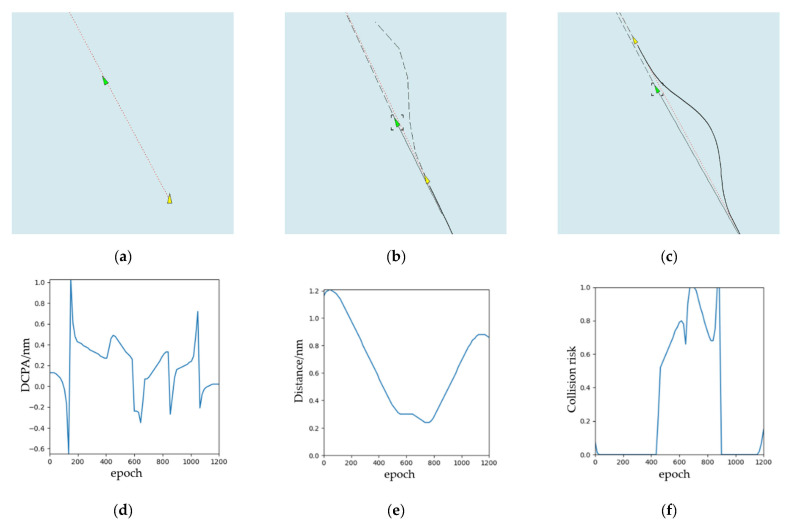
The experimental results (overtaking): (**a**) the start time during the collision avoidance process; (**b**) the middle time during the collision avoidance process; (**c**) the end time during the collision avoidance process; (**d**) DCPA; (**e**) distance; (**f**) collision risk.

The simulation results of overtaking scenario are shown in Figure 8. According to COLREGs Rule 13, the speed of the OS is larger than that of the TS. The relative position between this ship and other ships is 89° behind the right cross. From Figure 8a–c, this ship turns right to overtake and let other ships clear, which meets the requirements of the COLREGs. The ship turned twice in total. When OS bypasses TS, it will return to the original route. We can observe from Figure 8d,e, there are DCPA, distance from OS to TS, and risk of collision separately. It changes with the iterative training epoch-to-epoch.

### 5.2. Scenario 2: Head-On

In this simulation experiment, there is a target ship keeping direction and speed, and the OS needs to avoid the TS from reaching the target point. Similar to scenario 1 (Table 4), Table 4 shows the initial information of the OS and TS.

**Figure 9:**
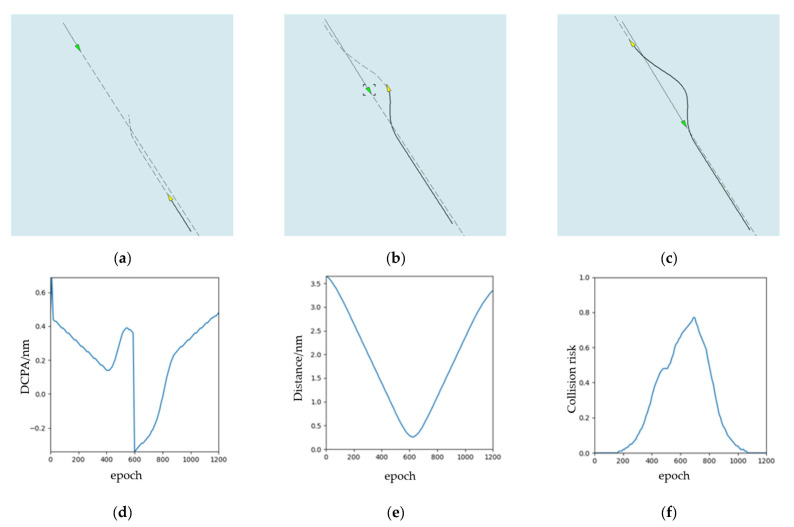
The experimental results (head-on): (**a**) the start time during the collision avoidance process; (**b**) the middle time during the collision avoidance process; (**c**) the end time during the collision avoidance process; (**d**) DCPA; (**e**) distance; (**f**) collision risk.

The experimental results are shown in Figure 9. From Figure 9a–c, we can see the status of OS and the collision avoidance trajectory in the start time, the middle time, and the end time. Following COLREGs Rule 14, when the course of two ships is opposite or close to the opposite, the head-on scenario is formed. Meanwhile, we can see that OS turns to the right at a large angle to avoid TS. The collision avoidance behavior of OS conforms to COLREGs. When OS bypasses TS, it will return to the original route. In addition, we can see from Figure 9d,e, there are DCPA, distance from OS to TS, and risk of collision shown separately. It changes epoch-to-epoch.

### 5.3. Scenario 3: Crossing

In this simulation experiment, we designed a crossing scenario with a small angle. There is a TS keeping direction and speed, and the OS needs to avoid the TS from reaching the target point. Table 5 shares the initial information of the OS and TS.

**Figure 10:**
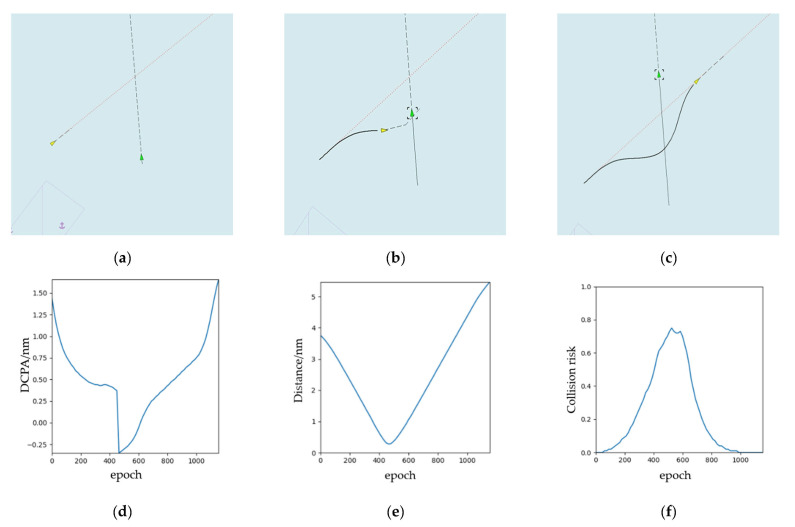
The experimental results (crossing): (**a**) the start time during the collision avoidance process; (**b**) the middle time during the collision avoidance process; (**c**) the end time during the collision avoidance process; (**d**) DCPA; (**e**) distance; (**f**) collision risk.

The experimental results are shown in Figure 10. Following COLREGs Rule 15, when two motorized vessels cross each other, causing a collision risk, the vessel with another vessel on the starboard side of the vessel shall give way to the other vessel. Therefore, the OS is the giving-way ship. From Figure 10a–c, we can see the status of the OS and the collision avoidance trajectory in the start time, the middle time, and the end time. Due to the open water navigation environment, the OS sails from the stern of the TS, and avoids crossing the front of the TS in accordance with COLREGs. When the OS bypasses TS, it will return to the original route. We can see from Figure 10d,e, the change of DCPA, the distance from the OS to TS, and risk of collision are shown separately.

### 5.4. Scenario 4: Multi-Ship Encounter into Open Water

Before this, we designed the most basic scenarios of two ships’ encounter, such as overtaking, head-on, and crossing. From the results, the navigation behavior and obstacle avoidance trajectory of the collision avoidance algorithm is consistent with the COLREGs. However, for the actual ship navigation, the most dangerous is the scenario of an encounter among multiple ships. Whether for the traditional manned ship or MASS, the rules do not limit the collision avoidance behavior when multiple ships encounter each other. This is also a key verification and performance test of the algorithm.

In this simulation experiment, we designed a multi-ship scenario—an encounter into open water. There are four target ships keeping direction and speed, and the OS needs to avoid these TSs from reaching the target point. Table 6 summarizes the initial information of the OS and the four TSs (TS 1 to TS 4).

**Figure 11:**
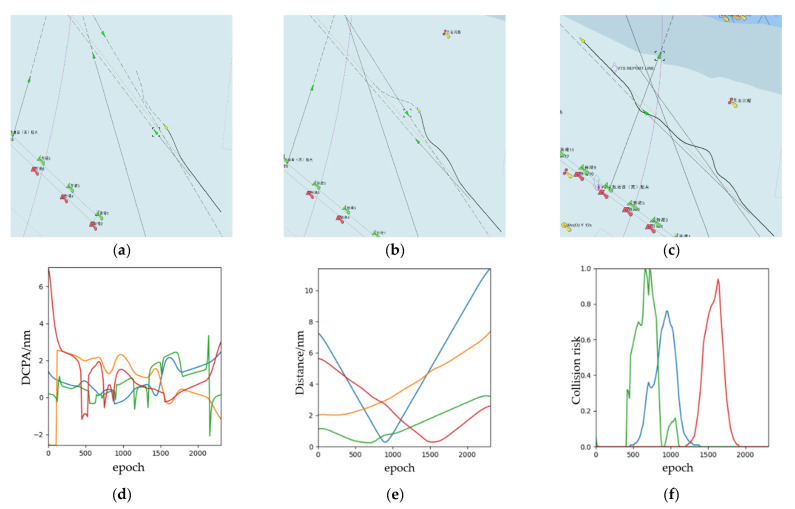
The experimental results (multi-ship encounter into open water): (**a**) the start time during the collision avoidance process; (**b**) the middle time during the collision avoidance process; (**c**) the end time during the collision avoidance process; (**d**) DCPA; (**e**) distance; (**f**) collision risk (Some subgraphs include some Chinese name and number information of navigation lights in channel, which is inherent in the system of ECDIS and has no influence on the conclusion of this paper).

The experimental results are shown in Figure 11. We find that in the navigation situation with four TSs, there are overtaking, head-on, and crossing encounter scenarios at the same time. From Figure 11a–c, we can see the status of the OS and the collision avoidance trajectory in the first collision avoidance time, the second collision avoidance time, and the arriving target point time. For the multi-ship collision avoidance problem in this experiment, the OS made three obstacle avoidance trajectory planning and collision avoidance decisions. The simulation results reveal that the OS can avoid all TS ships clearly, and the OS will return to the original route when the OS bypasses the TS. In the end, Figure 11d,e show that the change of DCPA, the distance from the OS to the TS, and the risk of collision, separately.

### 5.5. Scenario 5: Multi-Ship Encounter in a Busy Sea Area

As the busiest water area in the whole voyage, the channel entrance has the highest risk and the greatest dependence on the autonomous collision-avoidance navigation system. Different from the open waters, the ship collision avoidance at the channel entrance has more constraints. The simulation of the channel entrance environment can better verify the performance of the algorithm and the ability of autonomous collision avoidance.

In this case study section, we designed a scenario of a multi-ship encounter in a busy sea area. There are three target ships keeping direction and speed, and the OS needs to avoid these TSs and the entrance to the channel. For the navigation situation, we designed a ship that enters the traffic lane of the channel, a ship that exits the channel, and one ship that crosses the channel. Table 7 tabulates the initial information of the OS and the three TSs.

**Figure 12:**
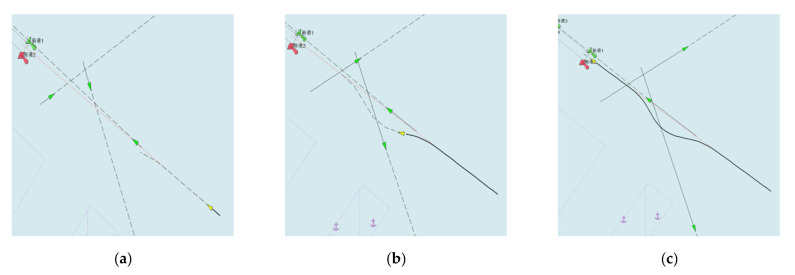

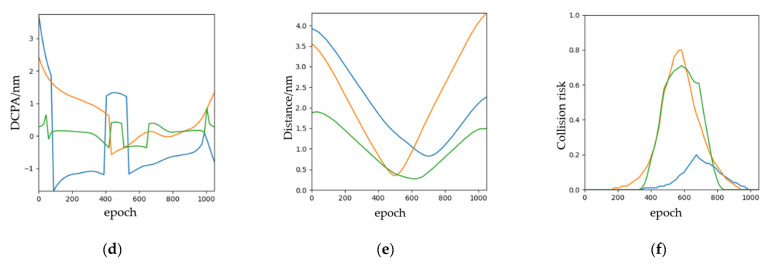
The experimental results (multi-ship encounter in a busy sea area): (**a**) the start time during the collision avoidance process; (**b**) the middle time during the collision avoidance process; (**c**) the end time during the collision avoidance process; (**d**) DCPA; (**e**) distance; (**f**) collision risk (Some subgraphs include some Chinese name and number information of navigation lights in channel, which is inherent in the system of ECDIS and has no influence on the conclusion of this paper).

The experimental results of multi-ship encounters in a busy sea area scenario are shown in Figure 12. From Figure 12a–c, we find that the OS can avoid the entering channel ship, exiting channel ship, and crossing channel ship one by one. There are overtaking scenarios, crossing with small-angle scenarios, and crossing with large-angle scenarios in this case study. Finally, the ship enters the channel collision-free. In the end, Figure 12d,e show the change of DCPA, the distance from the OS to TS, and the risk of collision, separately. In the process of avoiding the TS ships, the speed change is consistent with the collision avoidance decision made in the current situation, and the distance between each TS and its TS is greater than the radius of the ship’s safety field of the ship, while ensuring the shortest collision avoidance path. This confirms the effectiveness of the real-time collision avoidance framework.

## 6. Conclusions

In this paper, we proposed a real-time collision avoidance framework based on B-spline and optimal decoupling control. For the front-end, by limiting the displacement, velocity, and acceleration of the parameterized ship, the change of MASS motion can be described, and the method of separating the MASS from the TS by hyperplane is proposed to achieve the collision-avoidance trajectory planning. In the back end, formulating the optimization problem as the minimization of the total sailing time, the optimal decoupling control is adopted to optimize the path and speed of the MASS, and the shortest collision avoidance trajectory and optimal collision-avoidance control strategy are solved. In the future, we plan to develop a more reliable and safe collision avoidance system for MASS in an uncertain environment. In addition, human-MASS interaction will be a considerable development trend, which determines how to shore control centers interact with autonomous navigation systems in an emergency.

## Figures and Tables

**Table 1:** Definition of symbols.

Symbol	Meaning	Unit/Range
*RD*	Relative distance	n mile
*RO*	Relative orientation	[−π,π)
$Q_{r}$	Relative course	[−π,π)
$v_{r}$	Relative speed	knot
$D_{b}$	Safe distance	n mile
$T_{b}$	Safe time	h

**Table 2:** The expressions of physical quantities of various motion forms.

DOF	Translation Velocity	Angle	Angular Velocity	Force	Moment
$G_{x}$	u	ϕ,φ (roll angle)	p	X	K
$G_{y}$	v	θ (trim angle)	q	Y	M
$G_{z}$	w	δ (yaw angle)	r	Z	N

**Table 3:** Initial information (overtaking).

Ship List	Position	Course	Speed	RD	Bearing
OS	(38.84° N, 118.28° E)	329°	15 kn	0 nm	0
TS	(38.82° N, 118.30° E)	328°	7.5 kn	2.2 nm	328°

**Table 4:** Initial information (head-on).

Ship List	Position	Course	Speed	RD	Bearing
OS	(38.82° N, 118.30° E)	305.7°	15 kn	0 nm	0
TS	(38.87° N, 118.26° E)	150.0°	5 kn	3.7 nm	304.0°

**Table 5:** Initial information (crossing).

Ship List	Position	Course	Speed	RD	Bearing
OS	(38.82° N, 118.25° E)	51.3°	10 kn	0 nm	0
TS	(38.82° N, 118.26° E)	355.0°	10.5 kn	3.7 nm	93.0°

**Table 6:** Initial information (multi-ship encounter into open water).

Ship List	Position	Course	Speed	RD	Bearing
OS	(38.97° N, 118.15° E)	316.4°	15 kn	0 nm	0
TS 1	(38.87° N, 118.27° E)	315.0°	5 kn	1.1 nm	314.0°
TS 2	(38.84° N, 118.25° E)	340.0°	16 kn	2.0 nm	251.0°
TS 3	(38.88° N, 118.17° E)	20.0°	5 kn	5.6 nm	286.0°
TS 4	(38.95° N, 118.19° E)	145.0°	10 kn	7.3 nm	322.0°

**Table 7:** Initial information (multi-ship encounter in a busy sea area).

Ship List	Position	Course	Speed	RD	Bearing
OS	(38.81° N, 118.30° E)	316.4°	18 kn	0 nm	0
TS 1	(38.84° N, 118.23° E)	60.0°	5 kn	3.9 nm	296.0°
TS 2	(38.85° N, 118.24° E)	160.0°	8 kn	3.6 nm	311.0°
TS 3	(38.83° N, 118.27° E)	305.0°	3 kn	1.9 nm	3.5.0°

## Data Availability

The data presented in this study are available on request from the corresponding author. The data are not publicly available due to privacy.

## References

[1] Zhang X., Wang C., Jiang L., An L., Yang R. (2021). Collision-avoidance navigation systems for Maritime Autonomous Surface Ships: A state of the art survey. Ocean Eng..

[2] Yan X.-P., Wang S.-W., Ma F., Liu Y.-C., Wang J. (2020). A novel path planning approach for smart cargo ships based on anisotropic fast marching. Expert Syst. Appl..

[3] Liu Y., Song R., Bucknall R., Zhang X. (2019). Intelligent multi-task allocation and planning for multiple unmanned surface vehicles (USVs) using self-organising maps and fast marching method. Inf. Sci..

[4] Wang X., Feng K., Wang G., Wang Q. (2021). Local path optimization method for unmanned ship based on particle swarm acceleration calculation and dynamic optimal control. Appl. Ocean Res..

[5] Zhang X., Wang C., Liu Y., Chen X. (2019). Decision-making for the autonomous navigation of maritime autonomous surface ships based on scene division and deep reinforcement learning. Sensors.

[6] Xie S., Chu X., Zheng M., Liu C. (2020). A composite learning method for multi-ship collision avoidance based on reinforcement learning and inverse control. Neurocomputing.

[7] Liu Z., Zhang Y., Yu X., Yuan C. (2016). Unmanned surface vehicles: An overview of developments and challenges. Annu. Rev. Control.

[8] Wang C., Zhang X., Cong L., Li J., Zhang J. (2019). Research on intelligent collision avoidance decision-making of unmanned ship in unknown environments. Evol. Syst..

[9] Tam C., Bucknall R., Greig A. (2009). Review of collision avoidance and path planning methods for ships in close range encounters. J. Navig..

[10] Huang Y., Chen L., Chen P., Negenborn R.R., van Gelder P.H.A.J.M. (2020). Ship collision avoidance methods: State-of-the-art. Saf. Sci..

[11] Huang Y., Chen L., Van Gelder P. (2019). Generalized velocity obstacle algorithm for preventing ship collisions at sea. Ocean Eng..

[12] Wang T., Yan X., Wang Y., Wu Q. (2017). Ship domain model for multi-ship collision avoidance decision-making with COLREGs based on artificial potential field. TransNav Int. J. Mar. Navig. Saf. Sea Transp..

[13] Singh Y., Bibuli M., Zereik E., Sharma S., Khan A., Sutton R. (2020). A novel double layered hybrid multi-robot framework for guidance and navigation of unmanned surface vehicles in a practical maritime environment. J. Mar. Sci. Eng..

[14] Yu L., Wei Z., Wang Z., Hu Y., Wang H. (2017). Path optimization of AUV based on smooth-RRT algorithm. Proceedings of the 2017 IEEE International Conference on Mechatronics and Automation (ICMA).

[15] Heo Y.J., Chung W.K. RRT-based path planning with kinematic constraints of AUV in underwater structured environment. Proceedings of the 2013 10th International Conference on Ubiquitous Robots and Ambient Intelligence (URAI).

[16] Chiang H.T.L., Tapia L. (2018). COLREG-RRT: An RRT-based COLREGS-compliant motion planner for surface vehicle navigation. IEEE Robot. Autom. Lett..

[17] Wang L., Zhang Z., Zhu Q., Ma S. (2020). Ship Route Planning Based on Double-Cycling Genetic Algorithm Considering Ship Maneuverability Constraint. IEEE Access.

[18] Su C.M., Chang K.Y., Cheng C.Y. (2012). Fuzzy decision on optimal collision avoidance measures for ships in vessel traffic service. J. Mar. Sci. Technol..

[19] Chun D.-H., Roh M.-I., Lee H.-W., Ha J., Yu D. (2021). Deep reinforcement learning-based collision avoidance for an autonomous ship. Ocean Eng..

[20] Pinto M., Ferreira B., Sobreira H., Matos A., Cruz N. (2013). Spline navigation and reactive collision avoidance with colregs for asvs. Proceedings of the 2013 OCEANS-San Diego.

[21] Wang S., Zhang Y., Li L. (2020). A collision avoidance decision-making system for autonomous ship based on modified velocity obstacle method. Ocean Eng..

[22] Lyu H., Yin Y. (2019). COLREGS-constrained real-time path planning for autonomous ships using modified artificial potential fields. J. Navig..

[23] Chen G., Wu T., Zhou Z. (2021). Research on Ship Meteorological Route Based on A-Star Algorithm. Math. Probl. Eng..

[24] Lazarowska A. (2015). Ship’s trajectory planning for collision avoidance at sea based on ant colony optimisation. J. Navig..

[25] Ni S., Liu Z., Cai Y., Wang X. (2018). Modelling of ship’s trajectory planning in collision situations by hybrid genetic algorithm. Pol. Marit. Res..

[26] Ahn J.H., Rhee K.P., You Y.J. (2012). A study on the collision avoidance of a ship using neural networks and fuzzy logic. Appl. Ocean Res..

[27] Liu Y.H., Du X.M., Yang S.H., Yeung D.S., Liu Z.Q., Wang X.Z., Yan H. (2006). The Design of a Fuzzy-Neural Network for Ship Collision Avoidance. Advances in Machine Learning and Cybernetics.

[28] Liu R.W., Nie J., Garg S., Xiong Z., Zhang Y., Hossain M.S. (2020). Data-driven trajectory quality improvement for promoting intelligent vessel traffic services in 6G-enabled maritime IoT systems. IEEE Internet Things J..

[29] Zhao L., Roh M.I. (2019). COLREGs-compliant multiship collision avoidance based on deep reinforcement learning. Ocean Eng..

[30] Sawada R., Sato K., Majima T. (2021). Automatic ship collision avoidance using deep reinforcement learning with LSTM in continuous action spaces. J. Mar. Sci. Technol..

[31] Zaccone R. (2021). COLREG-Compliant Optimal Path Planning for Real-Time Guidance and Control of Autonomous Ships. J. Mar. Sci. Eng..

[32] Donnarumma S., Figari M., Martelli M., Zaccone R. (2020). Simulation of the Guidance and Control Systems for Underactuated Vessels. Lecture Notes in Computer Science (Including Subseries Lecture Notes in Artificial Intelligence and Lecture Notes in Bioinformatics).

[33] Choi Y., Kim D., Hwang S., Kim H., Kim N., Han C. (2017). Dual-arm robot motion planning for collision avoidance using B-spline curve. Int. J. Precis. Eng. Manuf..

[34] Gómez-Bravo F., Cuesta F., Ollero A., Viguria A. (2008). Continuous curvature path generation based on β-spline curves for parking manoeuvres. Robot. Auton. Syst..

[35] Śmierzchalski R., Saeed K., Pejaś J. (2005). Ships’ domains as collision risk at sea in the evolutionary method of trajectory planning. Information Processing and Security Systems.

[36] Fossen T.I. (2011). Handbook of Marine Craft Hydrodynamics and Motion Control.

